# Setting an agenda for comparative effectiveness systematic reviews in CKD care

**DOI:** 10.1186/1471-2369-13-74

**Published:** 2012-08-01

**Authors:** Deidra C Crews, Raquel C Greer, Jeffrey J Fadrowski, Michael J Choi, David Doggett, Jodi B Segal, Kemi A Fawole, Pammie R Crawford, L Ebony Boulware

**Affiliations:** 1 Division of Nephrology, Department of Medicine, Baltimore, MD, USA; 2 Welch Center for Prevention, Epidemiology and Clinical Research, Baltimore, MD, USA; 3 Division of General Internal Medicine, Department of Medicine, Baltimore, MD, USA; 4 Division of Pediatric Nephrology, Department of Pediatrics, Baltimore, MD, USA; 5 Johns Hopkins Evidence-based Practice Center, Baltimore, MD, USA; 6 Department of Epidemiology, Johns Hopkins Medical Institutions, Baltimore, Maryland, USA; 7 Johns Hopkins Bayview Medical Center, 301 Mason F. Lord Drive, Suite 2500, Baltimore, MD, 21224, USA

**Keywords:** Chronic kidney disease, Evidence-based practice, Health services research

## Abstract

Systematic reviews comparing the effectiveness of strategies to prevent, detect, and treat chronic kidney disease are needed to inform patient care. We engaged stakeholders in the chronic kidney disease community to prioritize topics for future comparative effectiveness research systematic reviews. We developed a preliminary list of suggested topics and stakeholders refined and ranked topics based on their importance. Among 46 topics identified, stakeholders nominated 18 as ‘high’ priority. Most pertained to strategies to slow disease progression, including: (a) treat proteinuria, (b) improve access to care, (c) treat hypertension, (d) use health information technology, and (e) implement dietary strategies. Most (15 of 18) topics had been previously studied with two or more randomized controlled trials, indicating feasibility of rigorous systematic reviews. Chronic kidney disease topics rated by stakeholders as ‘high priority’ are varied in scope and may lead to quality systematic reviews impacting practice and policy.

## Introduction

Evidence to inform the optimal care of patients at risk of chronic kidney disease (CKD) incidence or progression is greatly needed. Patients, clinicians, and policy makers [1-7] want evidence about effective strategies to: (1) prevent incident CKD, (2) improve the accuracy and timely detection of CKD, (3) treat CKD effectively to limit progression to end stage renal disease (ESRD), and (4) decrease morbidity from CKD-related comorbid illnesses. Comparative effectiveness research (CER) compares “the benefits and harms of various interventions and strategies for preventing, diagnosing, treating and monitoring health conditions in real-world settings.” [8] The American Recovery and Reinvestment Act (ARRA) of 2009 provided $1.1 billion for CER which was divided and disbursed to the National Institutes of Health, the Agency for Healthcare Research and Quality and the Office of the Secretary of Health and Human Services [9]. The Institute of Medicine was tasked to recommend national priorities for research questions to be addressed by CER and supported by ARRA funds. Among the Institute’s list of 100 initial priority topics, two addressed kidney disease [8].

A core research method of CER is systematic review and synthesis of existing literature, but this has not been widely employed to study CKD care. Findings from systematic reviews can help decision-makers draw conclusions about effective care strategies employed among heterogeneous populations and clinical settings. Systematic reviews also help to identify gaps in the existing evidence that need to be addressed by future primary research studies [10].

CER studies may be distinguished from other types of clinical research by their explicit goal of being highly responsive to priorities of community stakeholders (persons or groups who have a vested interest in a clinical decision and the evidence that supports that decision) who will use study findings to support their decisions about care [11]. Research funding agencies increasingly recommend formal solicitation of stakeholder input as an important initial step in designing CER studies. [11,12]. To date, there has been little effort to identify priorities for CER among stakeholders in the CKD community and to share this with the CKD community at large. We engaged stakeholders within the CKD community to identify and prioritize topics for future CER systematic reviews and to help set an agenda for future primary CER studies of CKD care.

## Methods

### General approach

The Johns Hopkins University Evidence-Based Practice Center (EPC) assembled our team under a contract from the Agency for Healthcare Research and Quality (AHRQ) of the Department of Health and Human Services. Our primary goal was to use the input of community stakeholders to identify topics for systematic reviews of primary literature comparing the effectiveness of strategies to prevent, detect, and treat CKD or its complications. We also sought to identify topics for future CER studies of CKD care where the performance of systematic reviews might not be currently feasible due to lack of sufficient primary literature to review. Our team had clinical expertise in pediatric nephrology, adult nephrology, and general internal medicine, and research expertise in CER, clinical epidemiology and prevention of CKD, clinician practice patterns in the care of patients with CKD, health disparities, and systematic review methodology.

**Figure 1:**
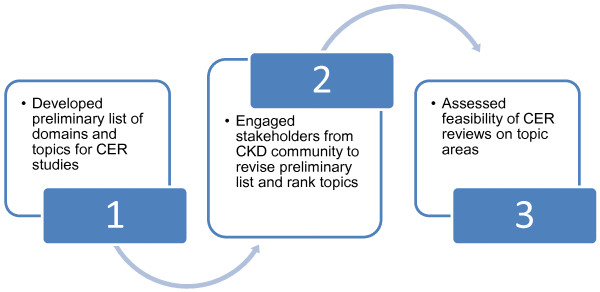
Chronic Kidney Disease Comparative Effectiveness Research (CER) Topic Identification Process.

We established a preliminary protocol for identifying and prioritizing topics, which we later revised with input from the stakeholders. The protocol consisted of three main activities: (1) identifying and categorizing potential topics of interest to the CKD community for systematic review and primary CER studies, (2) asking stakeholders to rank topics that would be of greatest interest to the CKD community, and (3) exploring the feasibility of performing CER systematic reviews on these high priority topics (Figure 1).

### Preliminary categorization and identification of topics for CER studies

From the outset, our team acknowledged the breadth of topics potentially relevant to the care of patients with CKD, ranging from studies of strategies to prevent CKD, to studies of strategies to prevent morbidity and mortality among patients with end stage renal disease (ESRD). We decided a priori to restrict our efforts to identifying priorities for CER reviews relevant to the care of patients with CKD stages I-IV because of time limitations (the process was funded to occur in less than 1 year) and differing considerations relevant to the care of patients with early or progressive CKD versus the care of patients with ESRD. To categorize the topics, we developed a preliminary ‘map’ reflecting broad domains of CKD care (prevention, detection, progression and complications) prior to engaging stakeholders. Within each broad domain, we developed a preliminary list of potential topics through an iterative process informed by our review of recent clinical practice guidelines [12-14] and perspectives pieces published by CKD thought leaders. We identified topics without regard to whether they were the subject of ongoing reviews. For many of the topics, we also identified numerous subtopics that could be of interest to the CKD community.

### Selection of CKD community stakeholder representatives

We sought to identify individuals representing varied stakeholder perspectives relevant to the performance of CER in CKD care. Many of the individuals we identified were employed or had active memberships in organizations representing relevant decision-makers in the CKD community and/or had clinical expertise relevant to the care of patients with CKD. (Table 1) Because we hypothesized government agencies such as the National Institutes of Health (NIH) might use the results of systematic reviews to guide funding decisions, we also engaged program officers overseeing clinical studies for their input.

**Table 1:** Organization Types and Clinical Expertise Represented by Expert Stakeholders*

**Organization Types**	**Clinical Expertise**
·Patient Advocacy Groups (2)	·Primary Care (Family Practice and Internal Medicine)
·Health Professional Societies (2)	·Adult Nephrology
·Private Healthcare Insurer (1)	·Pediatric Nephrology
·Governmental Healthcare Insurer (2)	·Endocrinology
·National Institutes of Health (2)	
Not representing an organization (2)	

* Stakeholders included 4 federal employees and 7 not affiliated with a governmental agency.

### Community Stakeholder Engagement

We asked stakeholders to provide input on our approach to identifying potential CER research topics and to rank the importance of topics. We engaged them via two conference calls and several electronic and mailed communications.

We reviewed definitions of CER as presented by AHRQ, the Institute of Medicine and the Federal Coordinating Council for Comparative Effectiveness Research [8] with stakeholders. We then presented the preliminary map of domains for CER topics to stakeholders. The map reflected four areas of potential interest to the CKD community: (1) prevention of CKD, (2) detection of CKD, (3) progression of CKD, and (4) complications of CKD. We also presented stakeholders with a preliminary list of 30 potential topics for CER research. We solicited stakeholders’ feedback regarding: (1) the relevance and appropriateness of our preliminary conceptual map, and (2) the relevance and comprehensiveness of the preliminary list of topics we proposed. We asked stakeholders to add to our preliminary list of topics.

We also asked stakeholders to establish a set of criteria for ranking topics. We proposed four criteria which they might consider, including (1) the *burden* of the topic to patients with CKD or patients at risk of developing CKD (including consideration of whether that topic was of particular relevance to priority subpopulations such as patients with diabetes, pediatric patients, racial/ethnic minorities or other vulnerable populations); (2) the extent to which there is *uncertainty* regarding optimal approaches to the topic; (3) the extent to which a systematic review with definitive findings could *impact* decision-making by patients, clinicians, or policy makers, and (4) the *feasibility* of performing a systematic review (i.e., adequacy of literature facilitating a rigorous review and the absence of recent previous systematic reviews). However, stakeholders expressed concern about the availability of literature to perform systematic reviews in several areas, and therefore decided to prioritize topics without regard to feasibility. Our team subsequently carried out a feasibility assessment of the stakeholder-prioritized topics.

Stakeholders revised our preliminary list of CER topics by refining the proposed topics and adding additional topics. Stakeholders then each independently ranked their 10 highest priority topics for CER studies (10 for highest, 1 for lowest). We calculated a summary score reflecting each topic’s overall standing among stakeholders (Additional file 1: Appendix A). We allowed ties for overall rankings and shared the final ranked list of topics with stakeholders to learn whether the composite rankings reflected the consensus of the entire group. We then asked them to identify topics they thought should be recommended for systematic reviews by AHRQ’s Evidence Based Practice Centers.

### Assessment of CER systematic review feasibility

We conducted preliminary literature searches to estimate the feasibility of performing CER systematic reviews on topics ranked highly by stakeholders. We searched publication titles in PubMed to identify relevant studies using terms sufficient to identify a reasonable core of the studies on a topic. We did not intend for these searches to be as exhaustive as would be required for formal systematic reviews. We assessed the availability of published systematic reviews/meta-analyses (within the past 5 years), randomized controlled trials (RCTs), and observational studies (if no systematic reviews/meta-analyses or RCTs were identified on a topic) for each top tier topic area. For RCTs and observational studies, we reviewed up to 300 of the most recent PubMed records for each topic. We noted whether studies pertained to specific priority populations and whether trial interventions were pharmacological or non-pharmacological. Search protocol and search terms are contained in Additional file 2: Appendix B.

## Findings

### Final categorization and list of potential topics for CER studies

Stakeholders agreed that the four proposed domains for categorizing topics for CER appropriately reflected the range of potential CER topics about CKD care, and added 16 additional topics to our preliminary list of 30 topics. Table 2 lists the 46 topics (grouped by domain, in no particular order) and example CER questions. The example questions are intended to illustrate ways in which CER topics could be framed, but they are not intended to limit the range of CER questions which could be asked regarding CER topic areas.

**Table 2:** List of topics considered by stakeholders for Comparative Effectiveness Systematic Reviews

	**Topic**	Example Question*
**Prevention**		
1**	Patient knowledge/education	What is the effectiveness of strategies to improve patient knowledge or awareness of CKD and CKD risk factors on decreasing CKD incidence?
**2**	Hypertension	What is the comparative effectiveness of hypertension management (pharmacologic and non-pharmacologic) on CKD incidence?
**3**	Diabetes control and prevention	What is the comparative effectiveness of diabetes management (pharmacologic and non-pharmacologic) on CKD incidence?
**4**	Patient Safety	What is the effectiveness of patient safety interventions on decreasing the incidence of acute kidney injury/CKD (i.e. exposure to contrast/dyes, nephrotoxins [NSAIDS])?
**5**	Smoking cessation and prevention	What is the comparative effectiveness of smoking cessation strategies on CKD incidence?
**6**	Lipid management	What is the comparative effectiveness of lipid management strategies on CKD incidence?
***7***	Vitamin D	What is the effectiveness of normalizing Vitamin D levels among deficient or insufficient patients in preventing CKD?
**8**	Obesity/Weight Management	What is the effectiveness of obesity/weight management interventions in decreasing CKD incidence?
**Detection**		
**9**	Screening benefits and harms	What are the benefits and harms of screening versus usual detection of CKD?
**10**	Screening frequency	What is the optimal screening frequency in populations at high-risk of CKD incidence?
**11**	Classification	Which estimations of GFR accurately classify people as having CKD? Including the correct stage?
**12**	Automated estimated GFR reporting	Does automated estimated GFR reporting lead to changes in clinical management and outcomes (drugs/referral)?
**Progression**		
**13**	Health Information Technology	What is the effectiveness of computer decision support for CKD management (including medication dosing) in slowing CKD progression?
**14**	Hyperuricemia	Is treatment of hyperuricemia an effective strategy for slowing CKD progression?
**15**	Inflammation	What is the comparative effectiveness of strategies to reduce markers of inflammation (i.e. C-reactive protein) in slowing CKD progression?
**16**	Provider awareness and guideline adherence	What is the effectiveness of strategies to improve provider awareness and adherence to guidelines on improving outcomes (progression) for patients with CKD?
**17**	Collaboration strategies	What is the effectiveness of strategies to increase collaboration in care (i.e. primary care/nephrology and team based approaches) of CKD patients in slowing CKD progression?
**18**	Diabetes management	What is the effectiveness and comparative effectiveness of diabetes management strategies (i.e. pharmacologic and behavioral strategies, therapeutic targets) in slowing CKD progression?
19	Vitamin D	Is vitamin D therapy effective in slowing CKD progression?
**20**	Hypertension	What is the comparative effectiveness of strategies to treat hypertension (i.e. target, medication combinations, behavioral strategies, lifestyle interventions) in slowing CKD progression?
**21**	Proteinuria	Is targeted therapy to reduce proteinuria (ie. optimal proteinuria target, specific therapies) effective in slowing CKD progression?
**22**	Patient safety	What is the effectiveness of patient safety interventions on slowing CKD progression?
**23**	Access to care	Is improved access to care (including primary or nephrology care) an effective mechanism for slowing CKD progression?
**24**	Cardiovascular disease	Are interventions to manage heart failure and coronary artery disease effective in slowing CKD progression?
**25**	Dietary strategies	What is the effectiveness or comparative effectiveness of dietary strategies in slowing CKD progression?
**26**	Congenital urologic disease	What is the effectiveness and comparative effectiveness of strategies to treat congenital urologic disease in slowing CKD progression?
**27**	Acute kidney injury	What is the comparative effectiveness of management strategies of AKI in slowing CKD progression?
**28**	Preparation for renal replacement therapy	What is the comparative of strategies (e.g., education, shared decision-making, fistula placement) to prepare patients for renal replacement therapy?
**29**	Metabolic acidosis	Is treatment of metabolic acidosis an effective strategy for slowing CKD progression?
**30**	Anemia	Is treatment of anemia an effective strategy for slowing CKD progression?
**31**	Dyslipidemia	Is treatment of dyslipidemia an effective strategy in slowing CKD progression?
**32**	Renovascular disease	Are renovascular interventions effective in slowing CKD progression?
**Complications**		
**33**	Cardiovascular Disease (CVD)	What is the effectiveness or comparative effectiveness of strategies to prevent or treat CVD among patients with CKD?
**34**	Patient Educational Interventions	What is the effectiveness of patient educational interventions in reducing CKD complications?
**35**	Bone/Mineral Disease	What is the effectiveness or comparative effectiveness of strategies to screen, evaluate and treat bone/mineral disease among patients with CKD?
**36**	Hypertension	What is the effectiveness or comparative effectiveness of blood pressure management strategies among patients with CKD?
**37**	Inflammation	What is the comparative effectiveness of strategies to reduce markers of inflammation (i.e., CRP) in slowing CKD progression?
**38**	Nutrition/Growth	What is the effectiveness of strategies to prevent, evaluate, and treat poor growth and malnutrition among patients with CKD?
**39**	Health Information Technology	What is the effectiveness of computer decision support for CKD management in reducing complications?
**40**	Collaborative Care	What is the effectiveness of strategies to increase collaboration (i.e., primary care/nephrology and team based approaches) in care of CKD for reducing CKD complications?
**41**	Patient Safety	What is the effectiveness of patient safety interventions in reducing complications among CKD patients?
**42**	Patient Reported Outcomes	What is the effectiveness of interventions to improve patient reported CKD complications (i.e. symptoms, sexual dysfunction, quality of life)?
**43**	Anemia	What is the effectiveness or comparative effectiveness of strategies to screen, evaluate, and treat anemia in patients with CKD?
**44**	Acute Kidney Injury	What is the effectiveness or comparative effectiveness of strategies to prevent or treat acute kidney injury among patients with CKD?
**45**	Functional status	What is the effectiveness of strategies to evaluate and improve functional status (i.e. rehabilitation interventions) among patients with CKD?
**46**	Fluid management	What is the effectiveness or comparative effectiveness of treatment strategies for volume overload among patients with CKD?

*Example questions are intended to illustrate ways in which CER topics can be framed, but are not intended to reflect questions themselves.

**Topics are grouped by domain, in no particular order.

### Final Stakeholder Prioritization of Topics for CER Studies

After stakeholders collectively reviewed the summary rankings, they agreed that topics with a global rank of 12 or better should be considered ‘high priority’ for future funded CER reviews. Because of ties, this cutoff resulted in 18 topics being considered ‘high priority.’ There was diversity in the opinions of the stakeholders regarding the appropriateness of topic areas for CER systematic reviews, with some stakeholders expressing concern regarding the likely paucity of evidence to compare existing strategies of care in certain topic areas. However, in general, stakeholders’ rankings were consistent, reflecting overall consensus regarding the appropriateness of topics for which CER systematic reviews would be of greatest interest to the CKD community. One anomaly was that one stakeholder’s top ranked topic, ‘inflammation in the progression of CKD’, was not ranked at all by any other stakeholder. Yet that top ranking by a single stakeholder was sufficient for it to be included among the top tier of topics. (Table 3) Half (n = 9) of top tier topics focused on strategies to slow or stop progression of CKD, while fewer top tier topics focused on prevention of CKD (n = 6), detection of CKD (n = 2), and complications of CKD (n = 1). Two topics emerged among the top tier priorities in more than one area of the conceptual map, patient safety (in prevention and progression areas) and hypertension (in prevention and progression areas).

**Table 3:** Topics receiving highest priority rankings for funded comparative Effectiveness Systematic Reviews in CKD

**Topic**	**CKD ** **Area**	Rank^*^	**Feasibility**			
			**Systematic reviews**	**Randomized controlled trials		
				**Pharmacologic**	**Behavioral**	**†Priority population**
Proteinuria	Progression	1	9	>100	20	0
Access to care	Progression	2	1	2	2	2
Hypertension	Progression	2	11	68	3	37
Screening benefits and harms	Detection	3	1	0	0	0
Hypertension control	Prevention	4	0	1	0	0
Patient knowledge/education	Prevention	5	0	0	3	3
Diabetes control and prevention	Prevention	6	2	18	3	23
Cardiovascular Disease	Complications	7	3	40	0	5
Patient safety	Progression	7	5	64	2	4
Health Information Technology	Progression	8	0	5	1	0
Patient Safety	Prevention	9	24	29	0	3
Vitamin D	Prevention	10	0	7	0	7
Classification	Detection	11	4	0	0	‡10
Obesity/Weight Management	Prevention	12	0	0	5	1
Dietary strategies	Progression	12	11	0	13	3
Inflammation	Progression	12	2	21	0	5
Collaboration strategies	Progression	12	0	0	2	1
Metabolic acidosis	Progression	12	0	4	0	0

* Topics with the same priority rank were tied in the overall score they received across all of the stakeholders. Rankings were based on responses received from 9 stakeholders.

**Randomized controlled trial categories are not mutually exclusive.

†Priority populations include: patients with diabetes, pediatric population, racial/ethnic minorities,

and other vulnerable populations.

‡Ten observational studies examined priority populations (27 total). No randomized control trials.

### Feasibility of performing CER systematic reviews on priority topics

Our preliminary assessment suggested some systematic reviews could be feasibly performed, with some topics having sufficient trial data and others having only observational data. (Table 3) Some of the priority topics identified, including ‘screening for CKD’ and ‘management of hypertension and lipid levels’, were previously reviewed in the AHRQ-funded evidence report *Screening for and Management of Chronic Kidney Disease Stages 1-3 *[15].

## Discussion

This project is one of the first attempts to systematically assess priorities for CER studies among stakeholders in the CKD community. Stakeholders’ high priority rankings reflect topics posing significant burden to patients with CKD or at risk of CKD, and aspects of CKD care for which there is significant uncertainty regarding effectiveness. High rankings also reflect stakeholders’ views that high-quality systematic reviews could help inform clinical practice or policy. While topics identified through this process may not include all topics that warrant CER systematic reviews or primary CER studies, this process may provide a preliminary road map for researchers seeking to perform CER studies relevant to the CKD community.

Many topics receiving a ‘high priority’ designation by stakeholders focused on studying the effectiveness of strategies to prevent CKD incidence and to slow CKD progression. Some highly ranked topic areas, such as ‘patient safety’, were reflected in multiple domains of the conceptual map. Because they are viewed as relevant to several aspects of CKD care and prevention, rigorous CER reviews on these topics could have high impact on patient care and clinical outcomes. Other topics, such as studies evaluating the comparative effectiveness of health information technology and the study of collaborative care strategies (both ranked as ‘top tier’ by our stakeholders), represent novel areas of inquiry. We identified few studies of these topics during our feasibility assessment, potentially reflecting a potential need for primary CER studies in these areas.

There are several caveats to our engagement of stakeholders for their input. First, the group of stakeholders was relatively small. Thus, individual stakeholders’ rankings could substantially affect topics’ overall final rankings and might not reflect fully the range of opinions that might be present among the entire CKD community. Indeed, some stakeholders did express concerns regarding the appropriateness of some topics for CER reviews, given a paucity of evidence to address these topics. Second, the composition of our stakeholder group and the manner in which we contacted stakeholders for participation in this exercise could also affect our findings. Third, while our stakeholder group included representatives from two patient advocacy organizations, we did not include actual patients with CKD. Methods for selecting patients with appropriate levels of expertise (i.e., medical knowledge, awareness of issues affecting patients with CKD relevant to comparative effectiveness research) for this type of scientific effort are not yet well defined. Fourth, stakeholders’ input may also have been further enriched by our inclusion of non-physician medical professionals such as nurses, social workers and dietitians. Future efforts to identify topics for CER reviews should consider broadening stakeholder representation to include patients and non-physician medical professionals. Fifth, some stakeholders did submit additional comments in with their rankings, which provided additional context to their rankings. However, because all stakeholders did not comment to the same extent, we did not formally analyze these comments. Finally, as this was one of the first funded efforts to identify priorities for CER systematic reviews in CKD, diversity in stakeholders’ opinions could also reflect their varying interpretations of the ultimate goal of activities such as this.

Other characteristics of our approach could also have influenced our findings. For instance, we developed the map and topic lists via iterative discussions prior to obtaining stakeholders’ input, and without regard to ongoing systematic reviews, which may have influenced the range of the final list of topics that the stakeholders ranked. Future projects of this kind may benefit from assessing and comparing independently determined priorities of different groups of stakeholders, including policy makers, funding agencies, clinicians and patients. Also, while our preliminary findings suggest several topics may have enough evidence to make CER systematic reviews feasible, several factors determine whether a systematic review will add value to inform medical decision-makers, including the number and quality of studies available to review, how definitively identified studies answer the question of interest, and how answers to questions will reduce uncertainty or add to the current knowledge and ultimately impact care. These factors should all be considered prior to undertaking CER systematic reviews.

In conclusion, we systematically identified priorities for CER research relevant to the care of patients with Stages I-IV CKD among stakeholders in the CKD community. Future efforts such as ours might benefit from identification of an even more broadly defined group of stakeholders (e.g., patients and non-physician CKD health professionals), further refinement of protocols for engaging stakeholders throughout the topic identification process, and development of strategies for identifying areas in need of both CER systematic reviews as well as primary CER studies. Findings from this project may be a useful guide for researchers and research funders seeking to address CER questions highly relevant to improving clinical care and outcomes of patients with CKD.

## Competing interests

There are no financial or non-financial competing interests amongst the authors.

## Authors’ contributions

DC participated in the design of the study, the preliminary identification of topics, and the feasibility assessment; and drafted the manuscript. RG, JF and MC each participated in the design of the study, the preliminary identification of topics, and the feasibility assessment; and helped to draft the manuscript. DD participated in the design and coordination of the study, the feasibility assessment and helped to draft the manuscript. JS participated in the design of the study and helped to draft the manuscript. KF and PC participated in the coordination of the study and the feasibility assessment. LEB oversaw all study activities, participated in the design of the study, participated in the preliminary identification of topics, conducted stakeholder engagement, and drafted the manuscript. All authors read and approved the final manuscript.

## Pre-publication history

The pre-publication history for this paper can be accessed here:

http://www.biomedcentral.com/1471-2369/13/74/prepub

## Supplementary Material

Additional file 1 **Appendix A.** Scoring protocol for topic prioritization. Click here for file

Additional file 2 **Appendix B.** Search Terms for Preliminary Feasibility Assessment on Each Topic. Click here for file
