# Supplementary material for: Setting an agenda for comparative effectiveness systematic reviews in CKD care
Source: BMC Nephrol. 2012 Aug 1;13:74. doi: 10.1186/1471-2369-13-74 (PMC3472164; doi:10.1186/1471-2369-13-74)
Supplement: Additional file 1 — Appendix A. Scoring protocol for topic prioritization. [file 1471-2369-13-74-S1.doc]

**Appendix A. Scoring protocol for topic prioritization**

1. Expert Stakeholder Representatives reviewed our populated framework and provided us with a list of their top 10 priority topics (in descending order).

2. We collected rankings and created a final ranked list of topics by the following method:

1. A score (1-10) was assigned to each topic for each Expert Stakeholder Representative as follows: Number 1 choice gets 10 points, number 2 gets 9 points,… etc. Scores were summed for each topic across all Representatives. Those topics with the highest scores were given the highest priority ranks. These global ranks ranged from 1 (highest score and highest priority) down to 20 (lowest ranked priority). Topics with tied scores received the same rank. Topics which received no score (not ranked in the top 10 of any Representatives) were unranked at the bottom of the global ranking.
2. This final global ranking was discussed in a telephone conference with the Expert Stakeholder Representatives.
