# Supplementary material for: Setting an agenda for comparative effectiveness systematic reviews in CKD care
Source: BMC Nephrol. 2012 Aug 1;13:74. doi: 10.1186/1471-2369-13-74 (PMC3472164; doi:10.1186/1471-2369-13-74)
Supplement: Additional file 2 — Appendix B. Search Terms for Preliminary Feasibility Assessment on Each Topic. [file 1471-2369-13-74-S2.doc]

**Appendix B. Search Terms for Preliminary Feasibility Assessment on Each Topic**

**Top Tier (priority 1-12)**

(Numbers in parenthesis are the stakeholder rankings)

**Limits for all topics:** Human, English

**Systematic Reviews and Meta-analyses:** All fields; no date limit

**RCTs:** Titles, unless fewer than 10 hits, then titles and abstracts; may be limited by date if there were substantially more than 300 hits

**Observational studies:** Titles; may be limited by date if there were substantially more than 300 hits; publication type filter - NOT (case reports[ptyp]) NOT (randomized controlled trial[ptyp]) NOT (systematic reviews[sb]) NOT meta-analysis[ptyp] NOT editorial[ptyp] NOT letter[ptyp] NOT (practice guideline[ptyp]) NOT review[ptyp] NOT (clinical conference[ptyp] NOT congresses[ptyp] NOT (consensus development conference[ptyp]) NOT comment[ptyp] NOT guideline[ptyp] NOT (interactive tutorial[ptyp]) NOT news[ptyp] NOT (newspaper article[ptyp]) NOT webcasts[ptyp]

**Prevention Domain**

**Hypertension control and prevention (4)**

(CKD OR "chronic kidney" OR "chronic renal" OR "kidney disease" OR "renal disease" OR "renal insufficiency" OR "kidney function" OR "kidney dysfunction" OR "renal function" OR "renal dysfunction" OR predialysis OR pre-dialysis OR nondialysis OR non-dialysis OR nondialyzed OR non-dialyzed OR nondialyzed OR non-dialyzed OR "glomerular filtration" OR "glomerular function" OR GFR OR proteinuria OR albuminuria OR creatinine) AND ("blood pressure" OR hypertension OR anti-hypertensive OR systolic OR diastolic OR "vascular resistance" OR "Beta blockers" OR "Beta blocker" OR "angiotensin converting enzyme inhibitors" OR "angiotensin converting enzyme inhibitor" OR "ACE Inhibitors" OR "ACE Inhibitor" OR "Calcium antagonist" OR "Calcium antagonists" OR "Arterial pressure" OR Renin-angiotensin OR "angiotensin receptor blockers" OR "angiotensin receptor blocker" OR ARB OR ARBs OR "calcium channel blockers" OR "calcium channel blocker" OR "aldosterone antagonists" OR "aldosterone antagonist" OR "alpha blockers" OR "alpha blocker" OR "loop diuretics" OR "loop diuretic" OR thiazide OR diuretics OR diuretic) AND (prevent OR prevents OR prevented OR prevention OR incidence OR incident OR “new case” OR initiate OR initiates OR initiated OR cause OR causes OR caused OR causation OR etiology OR predispose OR predisposes OR predisposed)

**Patient knowledge/patient education (5)**

Natural language search terms: (CKD OR "chronic kidney" OR "chronic renal" OR "kidney disease" OR "renal disease" OR "renal insufficiency" OR "kidney function" OR "kidney dysfunction" OR "renal function" OR "renal dysfunction" OR predialysis OR pre-dialysis OR nondialysis OR non-dialysis OR nondialyzed OR non-dialyzed OR nondialyzed OR non-dialyzed OR "glomerular filtration" OR "glomerular function" OR GFR OR proteinuria OR albuminuria OR microalbuminuria OR creatinine OR renoprotection OR reno-protection OR renoprotective OR reno-protective OR nephropathy OR nephropathic OR nephrology) AND (“Patient awareness” OR “awareness of patients” OR “patient knowledge” OR “knowledge of patients” OR “patient education” OR “education of patients” OR “patient teaching” OR “teaching patients” OR “teaching of patients”) AND (prevent OR prevents OR prevented OR prevention OR incidence OR incident OR “new case” OR initiate OR initiates OR initiated OR cause OR causes OR caused OR causation OR etiology OR predispose OR predisposes OR predisposed)

**Diabetes control and prevention (6)**

(CKD OR "chronic kidney" OR "chronic renal" OR "kidney disease" OR "renal disease" OR "renal insufficiency" OR "kidney function" OR "kidney dysfunction" OR "renal function" OR "renal dysfunction" OR predialysis OR pre-dialysis OR nondialysis OR non-dialysis OR nondialyzed OR non-dialyzed OR nondialyzed OR non-dialyzed OR "glomerular filtration" OR "glomerular function" OR GFR OR proteinuria OR albuminuria OR microalbuminuria OR creatinine)AND (diabetes OR diabetics OR diabetic OR glycation OR glycosylation OR glucose OR insulin OR "glycosylated hemoglobin" OR HbA1C OR "glucose control") AND (prevent OR prevents OR prevented OR prevention OR incidence OR incident OR “new case” OR initiate OR initiates OR initiated OR cause OR causes OR caused OR causation OR etiology OR predispose OR predisposes OR predisposed)

**Patient safety (9)**

(safety OR contrast OR dye OR dyes OR nephrotoxin OR nephrotoxins OR nephrotoxic OR “renal toxin” OR “renal toxins” OR renotoxic OR ((nonsteroidal OR non-steroidal OR nonsteroid OR non-steroid) AND (anti-inflamation OR anti-inflammatory))) AND (AKI OR “kidney injury” OR “renal injury” OR CKD OR "chronic kidney" OR "chronic renal" OR "kidney disease" OR "renal disease" OR "renal insufficiency" OR "kidney function" OR "kidney dysfunction" OR "renal function" OR "renal dysfunction" OR predialysis OR pre-dialysis OR nondialysis OR non-dialysis OR nondialyzed OR non-dialyzed OR nondialyzed OR non-dialyzed OR "glomerular filtration" OR "glomerular function" OR GFR OR proteinuria OR albuminuria OR microalbuminuria OR creatinine OR renoprotection OR reno-protection OR renoprotective OR reno-protective OR nephropathy OR nephropathic OR nephrology)

**Vitamin D (10)**

(CKD OR "chronic kidney" OR "chronic renal" OR "kidney disease" OR "renal disease" OR "renal insufficiency" OR "kidney function" OR "kidney dysfunction" OR "renal function" OR "renal dysfunction" OR predialysis OR pre-dialysis OR nondialysis OR non-dialysis OR nondialyzed OR non-dialyzed OR nondialyzed OR non-dialyzed OR "glomerular filtration" OR "glomerular function" OR GFR OR proteinuria OR albuminuria OR microalbuminuria OR creatinine OR renoprotection OR reno-protection OR renoprotective OR reno-protective OR nephropathy OR nephropathic OR nephrology) AND (Vitamin D OR Vitamin D2 OR Vitamin D3 OR 25(OH)D OR 1-alpha(OH)D3 OR 1,25(OH)2D3 OR alfacalcidol/calcitriol OR cholecalciferol OR calcidiol OR calcitriol OR doxercalciferol OR ergocalciferol OR paricalcitol OR dihydrotachysterol OR dihydroxycholecalciferol OR “vitamin D analog” OR “vitamin D analogue” OR “bone disease” OR osteoporosis OR fracture OR fractures) AND (prevent OR prevents OR prevented OR prevention OR incidence OR incident OR “new case” OR initiate OR initiates OR initiated OR cause OR causes OR caused OR causation OR etiology OR predispose OR predisposes OR predisposed)

**Obesity/weight management (12)**

((Obese OR Overweight OR “adiposity”) AND (diet OR exercise OR behavior OR lifestyle OR “antiobesity agents” OR Sibutramine OR Orlistat OR Phentermine OR Rimonabant)) AND (CKD OR "chronic kidney" OR "chronic renal" OR "kidney disease" OR "renal disease" OR "renal insufficiency" OR "kidney function" OR "kidney dysfunction" OR "renal function" OR "renal dysfunction" OR predialysis OR pre-dialysis OR nondialysis OR non-dialysis OR nondialyzed OR non-dialyzed OR nondialyzed OR non-dialyzed OR "glomerular filtration" OR "glomerular function" OR GFR OR proteinuria OR albuminuria OR microalbuminuria OR creatinine OR renoprotection OR reno-protection OR renoprotective OR reno-protective OR nephropathy OR nephropathic OR nephrology) AND (prevent OR prevents OR prevented OR prevention OR incidence OR incident OR “new case” OR initiate OR initiates OR initiated OR cause OR causes OR caused OR causation OR etiology OR predispose OR predisposes OR predisposed)

**Detection Domain**

**Screening benefits and harms (3) -** covered by the recently published AHRQ sys. rev. of CKD (Minnesota EPC); they identified no RCTs that compared systematic CKD screening versus no CKD screening, versus usual care, or versus an alternative CKD screening regimen and evaluated clinical outcomes.

**Classification (11)**

(CKD OR "chronic kidney" OR "chronic renal" OR "kidney disease" OR "renal disease" OR "renal insufficiency" OR "kidney function" OR "kidney dysfunction" OR "renal function" OR "renal dysfunction" OR predialysis OR pre-dialysis OR nondialysis OR non-dialysis OR nondialyzed OR non-dialyzed OR nondialyzed OR non-dialyzed OR "glomerular filtration" OR "glomerular function" OR GFR OR proteinuria OR albuminuria OR microalbuminuria OR creatinine OR renoprotection OR reno-protection OR renoprotective OR reno-protective OR nephropathy OR nephropathic OR nephrology) AND (classification OR classify OR classifying OR biomarker OR marker OR class OR stage OR staging OR MDRD OR “modification of diet in renal disease” OR Cockcroft-Gault OR CKD-EPI OR “chronic kidney disease epidemiology collaboration” OR “Mayo quadratic” OR Schwartz OR “Cystatin C” OR calibration OR “IDMS standard” OR “IDMS standardization”)

**Progression Domain**

**Proteinuria (1)**

(CKD OR "chronic kidney" OR "chronic renal" OR "kidney disease" OR "renal disease" OR "renal insufficiency" OR "kidney function" OR "kidney dysfunction" OR "renal function" OR "renal dysfunction" OR predialysis OR pre-dialysis OR nondialysis OR non-dialysis OR nondialyzed OR non-dialyzed OR nondialyzed OR non-dialyzed OR "glomerular filtration" OR "glomerular function" OR GFR OR creatinine) AND (proteinuria OR albuminuria OR microalbuminuria) AND (prevent OR prevents OR prevented OR prevention OR incidence OR incident OR “new case” OR initiate OR initiates OR initiated OR cause OR causes OR caused OR causation OR etiology OR predispose OR predisposes OR predisposed)

**Access to care (2)**

(CKD OR "chronic kidney" OR "chronic renal" OR "kidney disease" OR "renal disease" OR "renal insufficiency" OR "kidney function" OR "kidney dysfunction" OR "renal function" OR "renal dysfunction" OR predialysis OR pre-dialysis OR nondialysis OR non-dialysis OR nondialyzed OR non-dialyzed OR nondialyzed OR non-dialyzed OR "glomerular filtration" OR "glomerular function" OR GFR OR proteinuria OR albuminuria OR creatinine) AND ("access to care" OR referral OR insurance OR uninsured OR poverty OR disadvantaged OR socioeconomic OR "geographic region" OR distance OR "primary care provider" OR specialist OR Medicaid OR "Barriers to care")

**Hypertension (2)**

(CKD OR "chronic kidney" OR "chronic renal" OR "kidney disease" OR "renal disease" OR "renal insufficiency" OR "kidney function" OR "kidney dysfunction" OR "renal function" OR "renal dysfunction" OR predialysis OR pre-dialysis OR nondialysis OR non-dialysis OR nondialyzed OR non-dialyzed OR nondialyzed OR non-dialyzed OR "glomerular filtration" OR "glomerular function" OR GFR OR proteinuria OR albuminuria OR creatinine) AND ("blood pressure" OR hypertension OR anti-hypertensive OR systolic OR diastolic OR "vascular resistance" OR "Beta blockers" OR "Beta blocker" OR "angiotensin converting enzyme inhibitors" OR "angiotensin converting enzyme inhibitor" OR "ACE Inhibitors" OR "ACE Inhibitor" OR "Calcium antagonist" OR "Calcium antagonists" OR "Arterial pressure" OR Renin-angiotensin OR "angiotensin receptor blockers" OR "angiotensin receptor blocker" OR ARB OR ARBs OR "calcium channel blockers" OR "calcium channel blocker" OR "aldosterone antagonists" OR "aldosterone antagonist" OR "alpha blockers" OR "alpha blocker" OR "loop diuretics" OR "loop diuretic" OR thiazide OR diuretics OR diuretic) AND (prevent OR prevents OR prevented OR prevention OR incidence OR incident OR “new case” OR initiate OR initiates OR initiated OR cause OR causes OR caused OR causation OR etiology OR predispose OR predisposes OR predisposed)

**Patient safety (7)**

((safety OR contrast OR dye OR dyes OR nephrotoxin OR nephrotoxins OR nephrotoxic OR “renal toxin” OR “renal toxins” OR renotoxic OR ((nonsteroidal OR non-steroidal OR nonsteroid OR non-steroid) AND (anti-inflamation OR anti-inflammatory)))) AND (AKI OR “kidney injury” OR “renal injury” OR CKD OR "chronic kidney" OR "chronic renal" OR "kidney disease" OR "renal disease" OR "renal insufficiency" OR "kidney function" OR "kidney dysfunction" OR "renal function" OR "renal dysfunction" OR predialysis OR pre-dialysis OR nondialysis OR non-dialysis OR nondialyzed OR non-dialyzed OR nondialyzed OR non-dialyzed OR "glomerular filtration" OR "glomerular function" OR GFR OR proteinuria OR albuminuria OR microalbuminuria OR creatinine OR renoprotection OR reno-protection OR renoprotective OR reno-protective OR nephropathy OR nephropathic OR nephrology)

**Health information technology (8)**

(((shared AND "decision making" ) OR prescribing OR prescription OR prescriptions OR "disease management" OR "cognitive modeling" OR "patient care management" OR "care coordination" OR "shared decision making" OR communication OR "disease registry" OR "personal health record" OR "medical order" OR "medical record" OR "medical records" OR "self care" ) AND ("computer systems" OR computer OR tool OR electronic OR computerized OR "e-mail" OR "electronic mail" OR telemonitoring OR telemedicine OR "information technology" OR informatics OR "clinical decision support system" OR "natural language processing")) AND (CKD OR "chronic kidney" OR "chronic renal" OR "kidney disease" OR "renal disease" OR "renal insufficiency" OR "kidney function" OR "kidney dysfunction" OR "renal function" OR "renal dysfunction" OR predialysis OR pre-dialysis OR nondialysis OR non-dialysis OR nondialyzed OR non-dialyzed OR nondialyzed OR non-dialyzed OR "glomerular filtration" OR "glomerular function" OR GFR OR proteinuria OR albuminuria OR microalbuminuria OR creatinine OR renoprotection OR reno-protection OR renoprotective OR reno-protective OR nephropathy OR nephropathic OR nephrology)

**Dietary strategies (12)**

(CKD OR "chronic kidney" OR "chronic renal" OR "kidney disease" OR "renal disease" OR "renal insufficiency" OR "kidney function" OR "kidney dysfunction" OR "renal function" OR "renal dysfunction" OR predialysis OR pre-dialysis OR nondialysis OR non-dialysis OR nondialyzed OR non-dialyzed OR nondialyzed OR non-dialyzed OR "glomerular filtration" OR "glomerular function" OR GFR OR proteinuria OR albuminuria OR microalbuminuria OR creatinine OR renoprotection OR reno-protection OR renoprotective OR reno-protective OR nephropathy OR nephropathic OR nephrology) AND (diet OR dietary OR phosphate OR protein OR potassium OR nutrition OR sodium OR DASH OR Mediterranean OR "salt intake" OR "low fat" OR "fatty acid")

**Inflammation (12)**

(CKD OR "chronic kidney" OR "chronic renal" OR "kidney disease" OR "renal disease" OR "renal insufficiency" OR "kidney function" OR "kidney dysfunction" OR "renal function" OR "renal dysfunction" OR predialysis OR pre-dialysis OR nondialysis OR non-dialysis OR nondialyzed OR non-dialyzed OR nondialyzed OR non-dialyzed OR "glomerular filtration" OR "glomerular function" OR GFR OR proteinuria OR albuminuria OR microalbuminuria OR creatinine OR renoprotection OR reno-protection OR renoprotective OR reno-protective OR nephropathy OR nephropathic OR nephrology) AND (Inflammation OR inflammatory OR pro-inflammation OR pro-inflammatory OR c-reactive OR CRP OR TNF-alfa OR TNF-α OR “tumor necrosis factor-alpha” OR “tumor necrosis factor-α” OR cytokine OR cytokines OR oxidation OR oxidative OR “reactive oxygen” OR F2-isoprostane OR neutrophil OR neutrophils OR IL-1 OR interleukin-1 OR IL-1alpha OR IL-1α OR interleukin-1alpha OR interleukin-1α OR IL-1beta OR IL-1β OR interleukin-1beta OR interleukin-1β OR “IL-1 receptor antagonist” OR IL-1RN OR IL-1ra OR IL-6 OR interleukin-6 OR “plasma protein carbonyl” OR adiponectin OR “asymmetric dimethyl arginine” OR ADMA OR “toll-like receptor 9” OR TLR-9 OR oligodinucleotides OR ODN OR bacteria OR bacterial OR immune)

**Collaboration strategies (12)**

(collaboration OR collaborative OR collaborate OR coordination OR coordinate OR communication OR communicate OR “disease management” OR “care model” OR “care models” OR “care plan” OR “care plans” OR team OR teams OR multidisciplinary OR multidiscipline) AND (CKD OR "chronic kidney" OR "chronic renal" OR "kidney disease" OR "renal disease" OR "renal insufficiency" OR "kidney function" OR "kidney dysfunction" OR "renal function" OR "renal dysfunction" OR predialysis OR pre-dialysis OR nondialysis OR non-dialysis OR nondialyzed OR non-dialyzed OR nondialyzed OR non-dialyzed OR "glomerular filtration" OR "glomerular function" OR GFR OR proteinuria OR albuminuria OR microalbuminuria OR creatinine OR renoprotection OR reno-protection OR renoprotective OR reno-protective OR nephropathy OR nephropathic OR nephrology)

**Metabolic acidosis (12)**

(CKD OR "chronic kidney" OR "chronic renal" OR "kidney disease" OR "renal disease" OR "renal insufficiency" OR "kidney function" OR "kidney dysfunction" OR "renal function" OR "renal dysfunction" OR predialysis OR pre-dialysis OR nondialysis OR non-dialysis OR nondialyzed OR non-dialyzed OR nondialyzed OR non dialyzed OR "glomerular filtration" OR "glomerular function" OR GFR OR proteinuria OR albuminuria OR creatinine) AND (acidosis OR acidemia OR bicarbonate OR acid-base OR lactate OR “lactic acid” OR “serum pH” OR “hydrogen ion” OR “anion gap” OR potassium OR chloride OR “loop diuretics” OR furosemide)

**Complications Domain**

**Cardiovascular disease (7)**

(CKD OR "chronic kidney" OR "chronic renal" OR "kidney disease" OR "renal disease" OR "renal insufficiency" OR "kidney function" OR "kidney dysfunction" OR "renal function" OR "renal dysfunction" OR predialysis OR pre-dialysis OR nondialysis OR non-dialysis OR nondialyzed OR non-dialyzed OR nondialyzed OR non-dialyzed OR "glomerular filtration" OR "glomerular function" OR GFR OR proteinuria OR albuminuria OR microalbuminuria OR creatinine OR renoprotection OR reno-protection OR renoprotective OR reno-protective OR nephropathy OR nephropathic OR nephrology) AND (Cardiovascular OR “myocardial infarction” OR “coronary artery disease” OR “coronary heart disease” OR “ischemic heart disease” OR “acute coronary syndrome” OR stroke OR “cerebrovascular accident” OR “peripheral artery disease” OR claudication OR “peripheral arterial occlusive disease” OR “congestive heart failure” OR “heart failure”)

**Bottom Tier (priority <12)**

**Prevention Domain**

**Smoking cessation and prevention (16)**

Natural language search term: (CKD OR "chronic kidney" OR "chronic renal" OR "kidney disease" OR "renal disease" OR "renal insufficiency" OR "kidney function" OR "kidney dysfunction" OR "renal function" OR "renal dysfunction" OR predialysis OR pre-dialysis OR nondialysis OR non-dialysis OR nondialyzed OR non-dialyzed OR nondialyzed OR non-dialyzed OR "glomerular filtration" OR "glomerular function" OR GFR OR proteinuria OR albuminuria OR creatinine) AND (smoking OR smoker OR smokers OR nicotine OR tobacco OR cigarette OR cigarettes) AND (prevent OR prevents OR prevented OR prevention OR incidence OR incident OR “new case” OR initiate OR initiates OR initiated OR cause OR causes OR caused OR causation OR etiology OR predispose OR predisposes OR predisposed)

**Lipid management and prevention (20)**

((CKD OR "chronic kidney" OR "chronic renal" OR "kidney disease" OR "renal disease" OR "renal insufficiency" OR "kidney function" OR "kidney dysfunction" OR "renal function" OR "renal dysfunction" OR predialysis OR pre-dialysis OR nondialysis OR non-dialysis OR nondialyzed OR non-dialyzed OR nondialyzed OR non-dialyzed OR "glomerular filtration" OR "glomerular function" OR GFR OR proteinuria OR albuminuria OR microalbuminuria OR creatinine OR renoprotection OR reno-protection OR renoprotective OR reno-protective OR nephropathy OR nephropathic OR nephrology) AND (Lipid OR lipids OR Dyslipidemia OR Hyperlipidemia OR Hypercholesterolemia OR Statin OR Statins OR "HMG CoA reductase inhibitor" OR "HMG CoA reductase inhibitors" OR "cholesterol lowering" OR "lowering cholesterol" OR "lipid lowering" OR "lowering lipids" OR fibrate OR fibrates OR "bile acid sequestrants" OR "bile acid sequestrant" OR "cholesterol absorption inhibitors" OR "cholesterol absorption inhibitor") AND (prevent OR prevents OR prevented OR prevention OR incidence OR incident OR "new case" OR initiate OR initiates OR initiated OR cause OR causes OR caused OR causation OR etiology OR predispose OR predisposes OR predisposed) AND (Humans[Mesh] AND Randomized Controlled Trial[ptyp] AND English[lang])) NOT ((CKD OR "chronic kidney" OR "chronic renal" OR "kidney disease" OR "renal disease" OR "renal insufficiency" OR "kidney function" OR "kidney dysfunction" OR "renal function" OR "renal dysfunction" OR predialysis OR pre-dialysis OR nondialysis OR non-dialysis OR nondialyzed OR non-dialyzed OR nondialyzed OR non-dialyzed OR "glomerular filtration" OR "glomerular function" OR GFR OR proteinuria OR albuminuria OR creatinine) AND (Lipid OR lipids OR Dyslipidemia OR Hyperlipidemia OR Hypercholesterolemia OR Statin OR Statins OR "HMG CoA reductase inhibitor" OR "HMG CoA reductase inhibitors" OR "cholesterol lowering" OR "lowering cholesterol" OR "lipid lowering" OR "lowering lipids" OR fibrate OR fibrates OR "bile acid sequestrants" OR "bile acid sequestrant" OR "cholesterol absorption inhibitors" OR "cholesterol absorption inhibitor") AND (prevent OR prevents OR prevented OR prevention OR incidence OR incident OR "new case" OR initiate OR initiates OR initiated OR cause OR causes OR caused OR causation OR etiology OR predispose OR predisposes OR predisposed)

**Detection Domain**

**Screening frequency (13) -** covered by the recently published AHRQ sys. rev. of CKD (Minnesota EPC); they identified no RCTs that compared systematic CKD screening versus no CKD screening, versus usual care, or versus an alternative CKD screening regimen and evaluated clinical outcomes.

**Automated eGFR reporting (14)**

((CKD OR "chronic kidney" OR "chronic renal" OR "kidney disease" OR "renal disease" OR "renal insufficiency" OR "kidney function" OR "kidney dysfunction" OR "renal function" OR "renal dysfunction" OR predialysis OR pre-dialysis OR nondialysis OR non-dialysis OR nondialyzed OR non-dialyzed OR nondialyzed OR non-dialyzed OR "glomerular filtration" OR "glomerular function" OR GFR OR proteinuria OR albuminuria OR microalbuminuria OR creatinine OR renoprotection OR reno-protection OR renoprotective OR reno-protective OR nephropathy OR nephropathic OR nephrology) AND (eGFR OR "estimated glomerular filtration rate" OR "estimated GFR" OR GFR) AND (Humans AND Meta-Analysis AND English AND systematic)) NOT ((CKD OR "chronic kidney" OR "chronic renal" OR "kidney disease" OR "renal disease" OR "renal insufficiency" OR "kidney function" OR "kidney dysfunction" OR "renal function" OR "renal dysfunction" OR predialysis OR pre-dialysis OR nondialysis OR non-dialysis OR nondialyzed OR non-dialyzed OR nondialyzed OR non-dialyzed OR "glomerular function" OR proteinuria OR albuminuria OR creatinine) AND (eGFR OR "estimated glomerular filtration rate" OR "estimated GFR" OR GFR)

**Progression Domain**

**Anemia (15)**

((CKD OR "chronic kidney" OR "chronic renal" OR "kidney disease" OR "renal disease" OR "renal insufficiency" OR "kidney function" OR "kidney dysfunction" OR "renal function" OR "renal dysfunction" OR predialysis OR pre-dialysis OR nondialysis OR non-dialysis OR nondialyzed OR non-dialyzed OR nondialyzed OR non-dialyzed OR "glomerular filtration" OR "glomerular function" OR GFR OR proteinuria OR albuminuria OR microalbuminuria OR creatinine OR renoprotection OR reno-protection OR renoprotective OR reno-protective OR nephropathy OR nephropathic OR nephrology)) AND (anemia OR hemoglobin OR haemoglobin OR "erythropoiesis-stimulating" OR ESA OR hematopoietic OR epoetin OR darbepoetin OR hematocrit OR haematocrit)

**Diabetes management (16)**

(CKD OR "chronic kidney" OR "chronic renal" OR "kidney disease" OR "renal disease" OR "renal insufficiency" OR "kidney function" OR "kidney dysfunction" OR "renal function" OR "renal dysfunction" OR predialysis OR pre-dialysis OR nondialysis OR non-dialysis OR nondialyzed OR non-dialyzed OR nondialyzed OR non-dialyzed OR "glomerular filtration" OR "glomerular function" OR GFR OR proteinuria OR albuminuria OR microalbuminuria OR creatinine)AND (diabetes OR diabetics OR diabetic OR glycation OR glycosylation OR glucose OR insulin OR "glycosylated hemoglobin" OR HbA1C OR "glucose control")

**Congenital urologic disease (17)**

(CKD OR "chronic kidney" OR "chronic renal" OR "kidney disease" OR "renal disease" OR "renal insufficiency" OR "kidney function" OR "kidney dysfunction" OR "renal function" OR "renal dysfunction" OR predialysis OR pre-dialysis OR nondialysis OR non-dialysis OR nondialyzed OR non-dialyzed OR nondialyzed OR non-dialyzed OR "glomerular filtration" OR "glomerular function" OR GFR OR proteinuria OR albuminuria OR creatinine) AND ("Congenital urologic disease" OR CAKUT OR "congenital abnormalities of kidneys and urologic tract" OR "Obstructive uropathy" OR "Vesicoureteral reflux" OR "Urinary tract infection" OR UTI OR "Hydronephrosis" OR "Urethral valves" OR "Polycystic kidney")

**Preparation for renal replacement therapy (17) –** (renal OR kidney) AND (replacement OR transplantation OR transplant OR dialysis OR hemodialysis OR fistula) AND (preparation OR prepare OR planning OR plan OR decision OR decision-making OR education OR educate OR referral OR refer)

**Provider awareness and guideline adherence (19)**

(CKD OR "chronic kidney" OR "chronic renal" OR "kidney disease" OR "renal disease" OR "renal insufficiency" OR "kidney function" OR "kidney dysfunction" OR "renal function" OR "renal dysfunction" OR predialysis OR pre-dialysis OR nondialysis OR non-dialysis OR nondialyzed OR non-dialyzed OR nondialyzed OR non dialyzed OR "glomerular filtration" OR "glomerular function" OR GFR OR proteinuria OR albuminuria OR creatinine) AND (guideline OR guidelines) AND (awareness OR knowledge OR use OR application OR practice)

**Vitamin D (19)**

(CKD OR "chronic kidney" OR "chronic renal" OR "kidney disease" OR "renal disease" OR "renal insufficiency" OR "kidney function" OR "kidney dysfunction" OR "renal function" OR "renal dysfunction" OR predialysis OR pre-dialysis OR nondialysis OR non-dialysis OR nondialyzed OR non-dialyzed OR nondialyzed OR non-dialyzed OR "glomerular filtration" OR "glomerular function" OR GFR OR proteinuria OR albuminuria OR creatinine) AND (Vitamin D OR Vitamin D2 OR Vitamin D3 OR 25(OH)D OR 1-alpha(OH)D3 OR 1,25(OH)2D3 OR alfacalcidol/calcitriol OR cholecalciferol OR calcidiol OR calcitriol OR doxercalciferol OR ergocalciferol OR paricalcitol OR dihydrotachysterol OR dihydroxycholecalciferol OR “vitamin D analog” OR “vitamin D analogue” OR “bone disease” OR osteoporosis OR fracture OR fractures)

**Cardiovascular disease (19)**

Natural language search terms: (CKD OR "chronic kidney" OR "chronic renal" OR "kidney disease" OR "renal disease" OR "renal insufficiency" OR "kidney function" OR "kidney dysfunction" OR "renal function" OR "renal dysfunction" OR predialysis OR pre-dialysis OR nondialysis OR non-dialysis OR nondialyzed OR non-dialyzed OR nondialyzed OR non-dialyzed OR "glomerular filtration" OR "glomerular function" OR GFR OR proteinuria OR albuminuria OR microalbuminuria OR creatinine OR renoprotection OR reno-protection OR renoprotective OR reno-protective OR nephropathy OR nephropathic OR nephrology) AND (Cardiovascular OR “myocardial infarction” OR “coronary artery disease” OR “coronary heart disease” OR “ischemic heart disease” OR “acute coronary syndrome” OR stroke OR “cerebrovascular accident” OR “peripheral artery disease” OR claudication OR “peripheral arterial occlusive disease” OR “congestive heart failure” OR “heart failure”)

**Hyperuricemia (20)**

(CKD OR "chronic kidney" OR "chronic renal" OR "kidney disease" OR "renal disease" OR "renal insufficiency" OR "kidney function" OR "kidney dysfunction" OR "renal function" OR "renal dysfunction" OR predialysis OR pre-dialysis OR nondialysis OR non-dialysis OR nondialyzed OR non-dialyzed OR nondialyzed OR non dialyzed OR "glomerular filtration" OR "glomerular function" OR GFR OR proteinuria OR albuminuria OR creatinine) AND (hyperuricemia OR “uric acid”)

**Acute kidney injury (>20)**

(CKD OR "chronic kidney" OR "chronic renal" OR "kidney disease" OR "renal disease" OR "renal insufficiency" OR "kidney function" OR "kidney dysfunction" OR "renal function" OR "renal dysfunction" OR predialysis OR pre-dialysis OR nondialysis OR non-dialysis OR nondialyzed OR non-dialyzed OR nondialyzed OR non-dialyzed OR "glomerular filtration" OR "glomerular function" OR GFR OR proteinuria OR albuminuria OR microalbuminuria OR creatinine OR renoprotection OR reno-protection OR renoprotective OR reno-protective OR nephropathy OR nephropathic OR nephrology) AND ("Acute kidney injury" OR "Acute renal failure" OR "Acute renal insufficiency" OR "Acute tubular necrosis" OR "Renal ischemia" OR Oliguria OR Nephrotoxins OR Glomerulonephritis OR Nephritis)

**Dyslipidemia (>20)**

Natural language search terms: (CKD OR "chronic kidney" OR "chronic renal" OR "kidney disease" OR "renal disease" OR "renal insufficiency" OR "kidney function" OR "kidney dysfunction" OR "renal function" OR "renal dysfunction" OR predialysis OR pre-dialysis OR nondialysis OR non-dialysis OR nondialyzed OR non-dialyzed OR nondialyzed OR non-dialyzed OR "glomerular filtration" OR "glomerular function" OR GFR OR proteinuria OR albuminuria OR creatinine) AND (Dyslipidemia OR hyperlipidemia OR hypercholesterolemia OR statin OR "HMG CoA reductase inhibitor" OR "cholesterol lowering" OR "lipid lowering" OR fibrates OR "bile acid sequestrants" OR "cholesterol absorption inhibitors" OR anorexiants OR "lipase inhibitors")

**Renovascular disease (>20)**

(CKD OR "chronic kidney" OR "chronic renal" OR "kidney disease" OR "renal disease" OR "renal insufficiency" OR "kidney function" OR "kidney dysfunction" OR "renal function" OR "renal dysfunction" OR predialysis OR pre-dialysis OR nondialysis OR non-dialysis OR nondialyzed OR non-dialyzed OR nondialyzed OR non-dialyzed OR "glomerular filtration" OR "glomerular function" OR GFR OR proteinuria OR albuminuria OR creatinine) AND ("renal Perfusion" OR “percutaneous coronary” OR bypass OR “congestive heart” OR “renal artery” OR cardiorenal OR "Coronary revascularization" OR revascularization)

**Complications Domain**

**Health Information Technology (13)**

(((shared AND "decision making" ) OR prescribing OR prescription OR prescriptions OR "disease management" OR "cognitive modeling" OR "patient care management" OR "care coordination" OR "shared decision making" OR communication OR "disease registry" OR "personal health record" OR "medical order" OR "medical record" OR "medical records" OR "self care" ) AND ("computer systems" OR computer OR tool OR electronic OR computerized OR "e-mail" OR "electronic mail" OR telemonitoring OR telemedicine OR "information technology" OR informatics OR "clinical decision support system" OR "natural language processing")) AND (CKD OR "chronic kidney" OR "chronic renal" OR "kidney disease" OR "renal disease" OR "renal insufficiency" OR "kidney function" OR "kidney dysfunction" OR "renal function" OR "renal dysfunction" OR predialysis OR pre-dialysis OR nondialysis OR non-dialysis OR nondialyzed OR non-dialyzed OR nondialyzed OR non-dialyzed OR "glomerular filtration" OR "glomerular function" OR GFR OR proteinuria OR albuminuria OR microalbuminuria OR creatinine OR renoprotection OR reno-protection OR renoprotective OR reno-protective OR nephropathy OR nephropathic OR nephrology)

**Hypertension (14)**

(CKD OR "chronic kidney" OR "chronic renal" OR "kidney disease" OR "renal disease" OR "renal insufficiency" OR "kidney function" OR "kidney dysfunction" OR "renal function" OR "renal dysfunction" OR predialysis OR pre-dialysis OR nondialysis OR non-dialysis OR nondialyzed OR non-dialyzed OR nondialyzed OR non-dialyzed OR "glomerular filtration" OR "glomerular function" OR GFR OR proteinuria OR albuminuria OR creatinine) AND ("blood pressure" OR hypertension OR anti-hypertensive OR systolic OR diastolic OR "vascular resistance" OR "Beta blockers" OR "angiotensin converting enzyme inhibitors" OR "ACE Inhibitors" OR "Calcium antagonist" OR "Arterial pressure" OR Renin-angiotensin OR "angiotensin receptor blockers" OR ARB OR "calcium channel blockers" OR "aldosterone antagonists" OR "alpha blockers" OR "loop diuretics" OR thiazide OR diuretics)

**Bone/Mineral Disease (15)**

Natural language search terms: (CKD OR "chronic kidney" OR "chronic renal" OR "kidney disease" OR "renal disease" OR "renal insufficiency" OR "kidney function" OR "kidney dysfunction" OR "renal function" OR "renal dysfunction" OR predialysis OR pre-dialysis OR nondialysis OR non-dialysis OR nondialyzed OR non-dialyzed OR nondialyzed OR non-dialyzed OR "glomerular filtration" OR "glomerular function" OR GFR OR proteinuria OR albuminuria OR microalbuminuria OR creatinine OR renoprotection OR reno-protection OR renoprotective OR reno-protective OR nephropathy OR nephropathic OR nephrology) AND ("renal osteodystrophy" OR "secondary hyperparathyroidism" OR fracture OR "vascular calcification" OR "bone mineral metabolism" OR "adynamic bone disease" OR phosphate OR phosphorus OR calcium)

**Patient Educational Interventions (15)**

((health OR patient OR care) AND (education OR educational OR promotion OR promotional OR counseling OR counsel OR train OR training OR information OR instruction OR instructional)) AND (CKD OR "chronic kidney" OR "chronic renal" OR "kidney disease" OR "renal disease" OR "renal insufficiency" OR "kidney function" OR "kidney dysfunction" OR "renal function" OR "renal dysfunction" OR predialysis OR pre-dialysis OR nondialysis OR non-dialysis OR nondialyzed OR non-dialyzed OR nondialyzed OR non-dialyzed OR "glomerular filtration" OR "glomerular function" OR GFR OR proteinuria OR albuminuria OR microalbuminuria OR creatinine OR renoprotection OR reno-protection OR renoprotective OR reno-protective OR nephropathy OR nephropathic OR nephrology)

**Anemia (16)**

((CKD OR "chronic kidney" OR "chronic renal" OR "kidney disease" OR "renal disease" OR "renal insufficiency" OR "kidney function" OR "kidney dysfunction" OR "renal function" OR "renal dysfunction" OR predialysis OR pre-dialysis OR nondialysis OR non-dialysis OR nondialyzed OR non-dialyzed OR nondialyzed OR non-dialyzed OR "glomerular filtration" OR "glomerular function" OR GFR OR proteinuria OR albuminuria OR microalbuminuria OR creatinine OR renoprotection OR reno-protection OR renoprotective OR reno-protective OR nephropathy OR nephropathic OR nephrology)) AND (anemia OR hemoglobin OR haemoglobin OR "erythropoiesis-stimulating" OR ESA OR hematopoietic OR epoetin OR darbepoetin OR hematocrit OR haematocrit)

**Acute Kidney Injury (16)**

(CKD OR "chronic kidney" OR "chronic renal" OR "kidney disease" OR "renal disease" OR "renal insufficiency" OR "kidney function" OR "kidney dysfunction" OR "renal function" OR "renal dysfunction" OR predialysis OR pre-dialysis OR nondialysis OR non-dialysis OR nondialyzed OR non-dialyzed OR nondialyzed OR non-dialyzed OR "glomerular filtration" OR "glomerular function" OR GFR OR proteinuria OR albuminuria OR microalbuminuria OR creatinine OR renoprotection OR reno-protection OR renoprotective OR reno-protective OR nephropathy OR nephropathic OR nephrology) AND ("Acute kidney injury" OR "Acute renal failure" OR "Acute renal insufficiency" OR "Acute tubular necrosis" OR "Renal ischemia" OR Oliguria OR Nephrotoxins OR Glomerulonephritis OR Nephritis)

**Collaborative Care (18)**

(collaboration OR collaborative OR collaborate OR coordination OR coordinate OR communication OR communicate OR “disease management” OR “care model” OR “care models” OR “care plan” OR “care plans” OR team OR teams OR multidisciplinary OR multidiscipline) AND (CKD OR "chronic kidney" OR "chronic renal" OR "kidney disease" OR "renal disease" OR "renal insufficiency" OR "kidney function" OR "kidney dysfunction" OR "renal function" OR "renal dysfunction" OR predialysis OR pre-dialysis OR nondialysis OR non-dialysis OR nondialyzed OR non-dialyzed OR nondialyzed OR non-dialyzed OR "glomerular filtration" OR "glomerular function" OR GFR OR proteinuria OR albuminuria OR microalbuminuria OR creatinine OR renoprotection OR reno-protection OR renoprotective OR reno-protective OR nephropathy OR nephropathic OR nephrology)

**Patient Safety (18)**

((safety OR contrast OR dye OR dyes OR nephrotoxin OR nephrotoxins OR nephrotoxic OR “renal toxin” OR “renal toxins” OR renotoxic OR ((nonsteroidal OR non-steroidal OR nonsteroid OR non-steroid) AND (anti-inflamation OR anti-inflammatory)))) AND (AKI OR “kidney injury” OR “renal injury” OR CKD OR "chronic kidney" OR "chronic renal" OR "kidney disease" OR "renal disease" OR "renal insufficiency" OR "kidney function" OR "kidney dysfunction" OR "renal function" OR "renal dysfunction" OR predialysis OR pre-dialysis OR nondialysis OR non-dialysis OR nondialyzed OR non-dialyzed OR nondialyzed OR non-dialyzed OR "glomerular filtration" OR "glomerular function" OR GFR OR proteinuria OR albuminuria OR microalbuminuria OR creatinine OR renoprotection OR reno-protection OR renoprotective OR reno-protective OR nephropathy OR nephropathic OR nephrology)

**Nutrition/Growth (19)**

Natural language search terms: (CKD OR "chronic kidney" OR "chronic renal" OR "kidney disease" OR "renal disease" OR "renal insufficiency" OR "kidney function" OR "kidney dysfunction" OR "renal function" OR "renal dysfunction" OR predialysis OR pre-dialysis OR nondialysis OR non-dialysis OR nondialyzed OR non-dialyzed OR nondialyzed OR non-dialyzed OR "glomerular filtration" OR "glomerular function" OR GFR OR proteinuria OR albuminuria OR microalbuminuria OR creatinine OR renoprotection OR reno-protection OR renoprotective OR reno-protective OR nephropathy OR nephropathic OR nephrology) AND (nutrition OR nourishment OR nourished OR malnourishment OR malnourished OR undernourished OR under-nourished OR underweight OR wasting OR growth)

**Inflammation (>20)**

Natural language search terms: (CKD OR "chronic kidney" OR "chronic renal" OR "kidney disease" OR "renal disease" OR "renal insufficiency" OR "kidney function" OR "kidney dysfunction" OR "renal function" OR "renal dysfunction" OR predialysis OR pre-dialysis OR nondialysis OR non-dialysis OR nondialyzed OR non-dialyzed OR nondialyzed OR non-dialyzed OR "glomerular filtration" OR "glomerular function" OR GFR OR proteinuria OR albuminuria OR microalbuminuria OR creatinine OR renoprotection OR reno-protection OR renoprotective OR reno-protective OR nephropathy OR nephropathic OR nephrology) AND (Inflammation OR inflammatory OR pro-inflammation OR pro-inflammatory OR c-reactive OR CRP OR TNF-alfa OR TNF-α OR “tumor necrosis factor-alpha” OR “tumor necrosis factor-α” OR cytokine OR cytokines OR oxidation OR oxidative OR “reactive oxygen” OR F2-isoprostane OR neutrophil OR neutrophils OR IL-1 OR interleukin-1 OR IL-1alpha OR IL-1α OR interleukin-1alpha OR interleukin-1α OR IL-1beta OR IL-1β OR interleukin-1beta OR interleukin-1β OR “IL-1 receptor antagonist” OR IL-1RN OR IL-1ra OR IL-6 OR interleukin-6 OR “plasma protein carbonyl” OR adiponectin OR “asymmetric dimethyl arginine” OR ADMA OR “toll-like receptor 9” OR TLR-9 OR oligodinucleotides OR ODN OR bacteria OR bacterial OR immune)

**Fluid Management Strategies (>20)**

(CKD OR "chronic kidney" OR "chronic renal" OR "kidney disease" OR "renal disease" OR "renal insufficiency" OR "kidney function" OR "kidney dysfunction" OR "renal function" OR "renal dysfunction" OR predialysis OR pre-dialysis OR nondialysis OR non-dialysis OR nondialyzed OR non-dialyzed OR nondialyzed OR non-dialyzed OR "glomerular filtration" OR "glomerular function" OR GFR OR proteinuria OR albuminuria OR microalbuminuria OR creatinine OR renoprotection OR reno-protection OR renoprotective OR reno-protective OR nephropathy OR nephropathic OR nephrology) AND (“volume overload” OR hypervolemic OR euvolemic OR “fluid balance” OR “fluid management” OR “fluid accumulation” OR edema OR diuretic OR “water retention”)
